# Supplementary material for: Identification of photoperiod-induced specific miRNAs in the adrenal glands of Sunite sheep (Ovis aries)
Source: Front Vet Sci. 2022 Jul 22;9:888207. doi: 10.3389/fvets.2022.888207 (PMC9354845; doi:10.3389/fvets.2022.888207)
Supplement: Supplementary file 1 [file Data_Sheet_1.ZIP › Supplementary file/miRDeep2 file.zip!/miRDeep2 file/Know_Structure/SP42A2/oar-mir-107.pdf]

miRBase precursor : oar-mir-107
Total read count : 6904
oar-miR-107 read count : 6898
remaining reads : 6

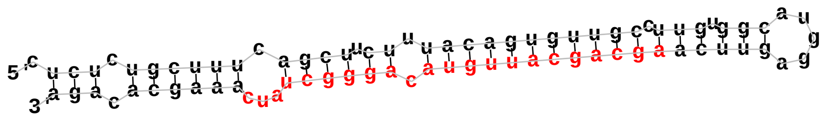

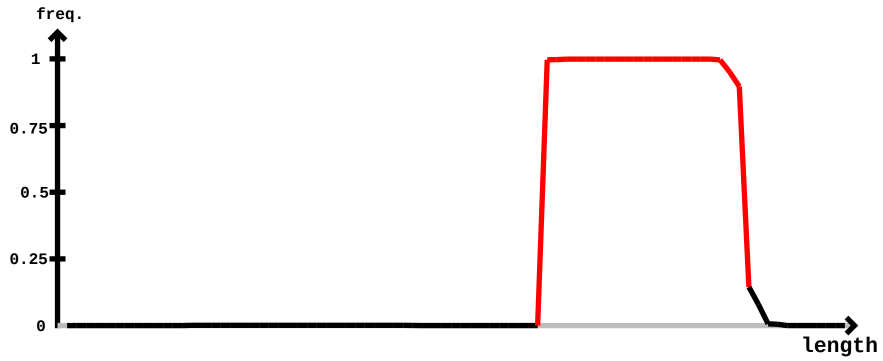

oar-miR-107

```
5'- cucucugcuuucagcuucuuuacaguguugccuugguggcauggaguucaagcagcauuguacagggcuaucaaagcacaga  -3'   exp
    .(((.((((((.((((.((.(((((((((((.(((.(((.....))))))))))))))))).))))))...)))))).)))   reads   mm   sample
    ...........agcuucuuuacaguguugccuug................................................   1       0    seq
    ...........agcuucuuuacagugCugccuug................................................   2       1    seq
    ...........agcuucuuuacagugCugccuugu...............................................   3       1    seq
    ..................................................agcagcauuguacagggc..............   21      0    seq
    ..................................................agcagcauuguacagggcu.............   311     0    seq
    ..................................................agcagcauuguacagggcua............   374     0    seq
    ..................................................agcagcauuguacagggcuau...........   5176    0    seq
    ..................................................agcagcauuguacagggcuauc..........   454     0    seq
    ..................................................agcagcauuguacagggcuauca.........   500     0    seq
    ..................................................agcagcauuguacagggcuaucaa........   12      0    seq
    ..................................................agcagcauuguacagggcuaucaaa.......   1       0    seq
    ..................................................agcagcauuguacagggcuauGaaa.......   32      1    seq
    ...................................................gcagcauuguacagggcuau...........   4       0    seq
    ....................................................cagcauuguacagggcu.............   1       0    seq
    ....................................................cagcauuguacagggcua............   2       0    seq
    ....................................................cagcauuguacagggcuau...........   8       0    seq
    ....................................................cagcauuguacagggcuauc..........   2       0    seq
```
